# A Preliminary Study of the Relationship between Promoter Methylation of the *ABCG1*, *GALNT2* and *HMGCR* Genes and Coronary Heart Disease

**DOI:** 10.1371/journal.pone.0102265

**Published:** 2014-08-01

**Authors:** Ping Peng, Lu Wang, Xi Yang, Xiaoyan Huang, Yanna Ba, Xiaoliang Chen, Jian Guo, Jiangfang Lian, Jianqing Zhou

**Affiliations:** 1 Ningbo Medical Center, Lihuili Hospital, Ningbo University, Ningbo, Zhejiang, China; 2 The Affiliated Hospital, School of Medicine, Ningbo University, Ningbo, Zhejiang, China; 3 School of Clinical Medicine, University of Cambridge, Cambridge, United Kingdom; University of North Carolina School of Medicine, United States of America

## Abstract

**Aims:**

To investigate the association of *ABCG1*, *GALNT2* and *HMGCR* genes promoter DNA methylation with coronary heart disease (CHD) and explore the interaction between their methylation status and the CHD patients' clinical characteristics in Han Chinese population.

**Methods and results:**

Methylation-specific polymerase chain reaction (MSP) technology was used to examine the role of the aberrant gene promoter methylation in CHD in Han Chinese population. A total of 85 CHD patients and 54 participants without CHD confirmed by angiography were recruited. 82.8% of the participants with *ABCG1* gene promoter hypermethylation have CHD, while only 17.4% of the participants without hypermethylation have it. The average age of the participants with *GALNT2* gene promoter hypermethylation is 62.10±8.21, while that of the participants without hypermethylation is 57.28±9.87; in the former group, 75.4% of the participants have CHD, compared to only 50% in the latter group. As for the *HMGCR* gene, the average age of the participants with promoter hypermethylation is 63.24±8.10 and that of the participants without hypermethylation is 57.79±9.55; its promoter hypermethylation is likely to be related to smoking. Our results indicated a significant statistical association of promoter methylation of the *ABCG1* gene with increased risk of CHD (OR = 19.966; 95% CI, 7.319–54.468; *P* ^*^<0.001; *P* ^*^: adjusted for age, gender, smoking, lipid level, hypertension, and diabetes). Similar results were obtained for that of the *GALNT2* gene (OR = 2.978; 95% CI, 1.335–6.646; *P* ^*^ = 0.008), but not of *HMGCR* gene (OR = 1.388; 95% CI, 0.572–3.371; *P* ^*^ = 0.469).

**Conclusions:**

The present work provides evidence to support the association of promoter DNA methylation status with the risk profile of CHD. Our data indicates that promoter DNA hypermethylation of the *ABCG1* and *GALNT2* genes, but not the *HMGCR* gene, is associated with an increased risk of CHD. CHD, smoking and aging are likely to be the important factors influencing DNA hypermethylation.

## Introduction

Coronary heart disease (CHD), a complex disease caused by an imbalance between blood supply and demand of the myocardium, is a leading cause of morbidity and mortality worldwide. The aetiology of CHD is largely attributed to the accumulation of cholesterol crystals, cell debris, fibrous materials, & minerals in the intimal layer of the coronary arteries [1]–[2]. It is well established that genetic and environmental factors, such as CHD family history, poor diet, advanced age, smoking habit, hypertension, diabetes mellitus, and dyslipidemia are associated with an increased risk of CHD [3]. Among these factors, dyslipidaemia is known to have a major influence. Several studies have suggested that an elevated level of total cholesterol (TC) and low-density lipoprotein cholesterol (LDL-C), and a low level of high density lipoprotein cholesterol (HDL-C) are associated with a worse prognosis of CHD [4]. Lipid accumulation product (LAP), a continuous marker of lipid over accumulation, has recently been proposed to be a good predictor for the risk of cardiovascular disease [5]. However, the exact role of dyslipidaemia in the cardiovascular system diseases remains to be determined.

Recently, increasing evidence indicates that many human diseases, including cancers and atherosclerosis, are either caused or influenced by abnormal DNA methylation [6]–[7]. DNA methylation is a stable and well understood epigenetic marker. It refers to the addition of a methyl group to the 5 position of cytosine in a dinucleotide CpG site. Although controversy still exists about the effect of the position and size of methylated DNA segments on regional transcription, it has been well established that this epigenetic change is associated with transcription silencing, and loss of methylation (demethylation) promotes, if not activates, gene expression [8]. One of the major mechanisms to down-regulate expression of a gene is methylation of a cytosine and guanine rich area, called CpGislands, in the promoter region of the gene [9]. A few studies have indicated that DNA promoter methylation of certain genes is responsible for the susceptibility of CHD [10]–[13].

*ABCG1*, *GALNT2* and *HMGCR* are among the genes suggested by Genome-wide association study (GWAS) whose variants are associated with CHD and variations in plasma lipoproteins [14]–[16]. These three genes exert their effects on the pathogenesis and progression of CHD through manipulating the various lipid pathways. The expression of *ABCG1* gene reduces cholesterol accumulation in macrophages by promoting the transfer of intracellular cholesterol into HDL-C pathway [15]. *GALNT2* gene, which influences O-linked oligosaccharide biosynthesis, has been shown to be involved in HDL-C regulation in humans [14], [16]. *HMGCR* gene, on the other hand, is associated with variation in LDL-C levels [14]. The aims of the present study are firstly to establish whether abnormal promoter region methylation of these three genes occurs in the cardiovascular system; secondly to explore the interaction of the methylation with the participants' lipid level and a few clinical characteristics; and thirdly to ascertain whether the aforementioned methylation contributes to the risk of CHD.

## Materials and Methods

### Ethics Statement

This study protocol was approved by the Ethical Committee of Ningbo Lihuili Hospital. The informed written consent was obtained from all the subjects.

### Sample and clinical data

Between 1st of July 2012 and 1st of March 2013,221 patients were under the care of our Cardiology department in the Ningbo Lihuili Hospital, Ningbo city of Zhejiang province, China. Each of them was seen and reviewed by at least two independent cardiologists. All of their medical records were carefully read. Among these patients, 85 patients had one or more major coronary arteries with more than 50% occlusion. They were included into the case group. Another 54 patients, who do not have an occlusion at all or a past medical history of any congenital heart disease, cardiomyopathy, liver or renal diseases, are included in the non-CHD group. The rest 82 patients were excluded from our study because of the existence of occlusion of less than 50% in at least one of their coronary arteries, or their past medical history of the conditions mentioned above. The details of the inclusion criteria were presented in our previous publication [17]. All individuals are Han Chinese living in Ningbo city for at least three generations. Blood samples were collected in 3.2% citrate sodium-treated tubes and then stored at −80°C.

### Biochemical analyses

Genomic DNA was isolated from peripheral blood samples using the Wizard Genomic DNA Purification kit (Promega, Madison, USA). DNA concentrations were determined by the ultramicro nucleic acid ultraviolet tester (NANODROP 1000, Wilmington, USA). Plasma levels of TG, TC, HDL, and LDL were measured using an enzymatic end point assay [18]. The ApoA, ApoB and ApoE levels were measured by the transmission turbidimetric method [19]. The plasma Lp(a) concentrations were determined by a sandwich enzyme-linked immunosorbent assay method [Macra-Lp(a), SDI, Newark, Delaware]. The concentrations of ALT, AST, ALP and GGT in plasma were measured by the IFCC reference measurement systems ([20]–[22]. The ALB level was worked through the Bromocresol green method [23]. All the above mentioned procedures were performed following the standard procedures recommended by the manufacturers.

### Epigenetic analysis

#### Sodium Bisulfite Conversion

The methylation status of a DNA sequence was measured using sodium bisulfite technology. Sodium bisulfite preferentially deaminatesun methylated cytosine residues to thymines (after PCR amplification), whereas methyl-cytosines remain unmodified. Therefore, bisulfite treatment introduces a difference to the DNA sequence of methylated and unmethylated DNA. Sodium bisulfite conversion and DNA recovery were performed by using EpiTect Bisulfite Kit (Qiagen) as follows: dilute a total of 2 µg DNA from blood in 40 µL of RNase-free water in a 200 µL PCR tube; add 85 µL Bisulfite Mix and 15 µL DNA Protect Buffer into the tube; and perform the bisulfite DNA conversion using a thermal cycler. DNA was denaturated at 95°C for 5 minutes, incubated at 60°C for 25 minutes, 95°C for 5 minutes, 60°C for 85 minutes, 95°C for 5 minutes, and 60°C for 175 minutes, and finally held at 20°C indefinitely. After bisulfite treatment, DNA was ethanol-precipitated and resuspended in 39 µ Lelution buffer (Buffer EB) and stored at −20°C.

#### Methylation – Specific PCR (MSP)

Methylation in the target CpG regions of *ABCG1*, *GALNT2* and *HMGCR* genes promoter in the bisulfite-modified DNA was investigated by methylation-specific PCR (MSP). Each PCR reaction mix has a total volume of 50 µL which contains 2 µL bisulfite-modified DNA, 10 µL 1× KAPA2G buffer (KAPA BIO, United States), 1 µL 0.2 mmol/L deoxynucleotide triphosphate mix (dNTP) mix (KAPA BIO, United States), 1 µL 0.5 µmol/L of each primer, and 0.5 U of KAPA2G Robust Hotstart DNA Polymerase (KAPA BIO, United States). The thermo-cyclere condition is as follows: (1) 95°C for 5 minutes; (2) 10 cycles of 95°C for 30 seconds, Tm-0.8°C for 30 seconds, and 72°C for 60 seconds; (3) 38 cycles of 95°C for 30 seconds, Tm°C for 30 seconds, and 72°C for 60 seconds; (4) 72°C for 10 minutes. PCR products were then electrophoresed on a 2.5% agarosegel and visualized under UV illumination. PCR primers were designed by Primer Express Software v2.0 (ABI). The gene names, locations, primer and probe sequences are summarized in Table 1.

**Table 1:** Primers for MSP of the *ABCG1*, *GALNT2* and *HMGCR* genes.

Group	Forward primer(5′to3′)	Reversr primer(5′to3′)	Product size (bp)	Tm
ABCG1 **M**	5′ ATTTGTATTGTGATATCGACGAGAC 3′	5′ CTTACCTCCTCGATTCTAAACGTAC 3′	251	54
ABCG1 **U**	5′ AGATTTGTATTGTGATATTGATGAGAT 3′	5′ AACTTACCTCCTCAATTCTAAACATAC 3′	251	48
GALNT2 **M**	5′ TTATAAGATAGATCGTTTTTTTGTATC 3′	5′ CCGCTAATATCGATTTTATTTAT 3′	263	48
GALNT2 **U**	5′ ATGTTATAAGATAGATTGTTTTTTTGTATT 3′	5′ AACCCACTAATATCAATTTTATTTAT 3′	263	46
HMGCR **M**	5′ TATAAGAGAGAGAGACGTAGGTGATC 3′	5′ CCCGTACTCGTCCTAACTATAATAA 3′	290	55
HMGCR **U**	5′ GTATATAAGAGAGAGAGATGTAGGTGATT 3′	5′ TAACCCATACTCATCCTAACTATAATAA 3′	290	54

MSP: methylation-specific polymerase chain reaction; M: methylation-specific primers; U: unmethylation-specific primers.

### Statistical analysis

Statistical analyses were performed using the PASW Statistics13.0 software (SPSS, Inc., Somers, NY, USA) and Graph Pad (Prism 5). Data are presented as means ± standard deviation. The differences of the *ABCG1*, *GALNT2* and *HMGCR* genes promoter methylation status between the CHD patients and the control group were analyzed using a Pearson Chi-square exact test. The mean group differences for laboratory parameters were compared by using a Student t-test. Pearson correlation was used to determine the association between the three genes promoter methylation status and CHD by assessing odds ratio (ORs) and 95% confidence intervals (95% CI). Cox regression was used to assess the effect of baseline and traditional risk factors, such as age, gender, smoking, lipid level, hypertension, and diabetes. All statistical analyses were two-sided and *P* value<0.05 was considered to be statistically significant.

## Results

The main clinical and biochemical characteristics of the study population are shown in table 2. There are 85 CHD patients with a mean age of 61.33±9.22 and 54 healthy subjects with a mean age of 56.35±9.00 participating in the present study. The CHD patients are older than the non-CHD subjects. There are more males present in the CHD group than in the non-CHD group. When all the participants are analysed according to their genders, the males' mean age is 59.22±9.52 years old, similar to that of the females, 59.73±9.34 years old. The percentage of smokers is higher in the male group compared to in the female group (66.3% vs. 9.8%, P<0.001).

**Table 2:** Characteristics of all subjects according to subgroup analysis by CHD status and gender.

Characteristics	Subgroup analysis by CHD status			Subgroup analysis by gender		
	CHD(n = 85)	Non-CHD(n = 54)	P value	Male(n = 89)	Female(n = 50)	P value
	(Mean ± s.e.)	(Mean ± s.e.)		(Mean ± s.e.)	(Mean ± s.e.)	
Age(yrs)	61.33±9.22	56.35±9.00	**0.002**	59.22±9.52	59.73±9.34	0.763
Gender (M/F)	(58/67.4)	(31/57.4)	0.230	NA	NA	
Smoking,n(%)	43(50.0)	21(38.9)	0.199	59(66.3)	5(9.8)	**<0.001**
Hypertension,n(%)	53(61.6)	26(48.1)	0.117	55(61.8)	24(47.1)	0.091
Diabetes,n(%)	25(29.1)	3(5.6)	**0.001**	15(16.9)	13(25.5)	0.219
TC(mmol/l)	4.5±1.1	4.35±1.0	0.399	4.48±1.09	4.38±1.02	0.591
TG(mmol/l)	1.97±2.82	1.47±0.72	0.203	1.92±2.76	1.53±0.90	0.323
HDL-C(mmol/l)	1.09±0.26	1.10±0.36	0.784	1.08±0.32	1.12±0.28	0.397
LDL-C(mmol/l)	2.72±0.92	2.63±0.76	0.547	2.74±0.88	2.60±0.83	0.388
ApoAI(g/L)	1.07±0.19	1.04±0.23	0.320	1.077±0.22	1.03±0.18	0.142
ApoB(g/L)	0.85±0.27	0.84±0.21	0.830	0.85±0.24	0.82±0.26	0.475
ApoE(g/L)	4.18±1.34	3.96±1.42	0.369	3.90±1.18	4.43±1.60	**0.026**
Lp(a)(g/L)	0.40±0.34	0.25±0.26	**0.005**	0.32±0.35	0.39±0.26	0.190
hs-CRP (mg/L)	8.09±14.81	3.90±3.87	**0.014**	7.63±14.55	4.47±4.62	0.134
ALB (g/L)	41.27±4.25	40.66±6.27	0.498	41.02±5.95	41.06±3.20	0.958
GLB (g/L)	24.40±3.60	26.26±4.94	**0.011**	24.98±4.37	25.37±4.05	0.597
A/G	1.72±0.32	1.64±0.29	0.121	1.71±0.33	1.65±0.28	0.239
ALT (IU/L)	27.06±18.19	24.94±17.20	0.496	29.01±19.65	21.41±12.72	**0.014**
AST (IU/L)	32.95±47.19	27.96±21.09	0.466	35.07±47.33	23.98±15.85	0.108
ALP (IU/L)	72.74±20.33	70.22±24.69	0.512	70.75±21.22	73.54±23.57	0.472
GGT (IU/L)	40.19±40.01	33.81±27.74	0.307	44.88±39.16	25.25±24.82	**<0.001**

Values are given as mean±s.e.; NA: denotes not applicable.

CHD: coronary heart disease; TC: total cholesterol; HDL: high density lipoprotein; LDL: low density lipoprotein; ALT: alanine aminotransferase; AST: aspartate aminotransferase.

**Figure 1:**
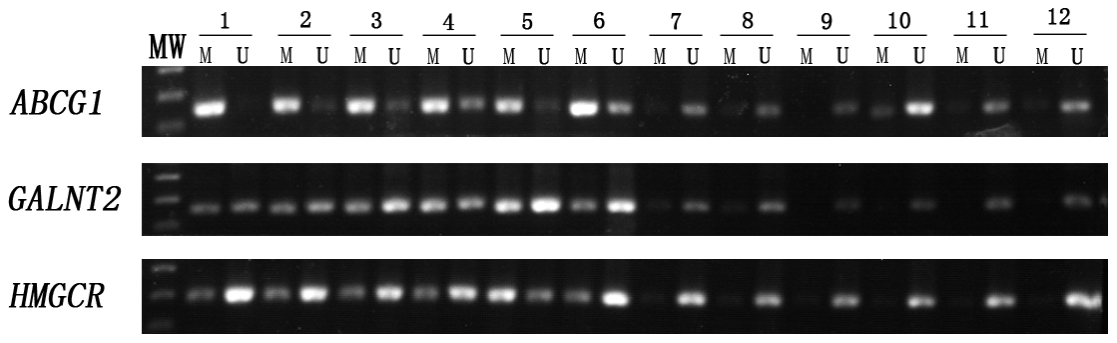
Typical methylation analysis result of the *ABCG1*, *GALNT2* and *HMGCR* genes promoter regions of by MSP. MW: molecular weight DNA marker (100-bp DNA ladder). 1–6 were CHD patients, 7–12 were Non-CHD controls; M for methylated specific primers; U for the unmethylated specific primers; PCR product indicated the presence of methylated or unmethylated promoter of *ABCG1*, *GALNT2* and *HMGCR* for primer M or primer U.

Fig. 1 shows a typical example of the MSP products analysed on an agarose gel for the *ABCG1*, *GALNT2* and *HMGCR* genes. We analysed the relationship between the promoter methylation status of the three genes and various clinical characteristics, such as age, sex, smoking, lipid level, hypertension, and diabetes mellitus in Table 3. Firstly, we found that 26.9% of the participants with *ABCG1* gene promoter hypermethylation have diabetes, while only 6.5% of the participants without hypermethylation have it. This difference is statistically significant. However, when the Cox regression was used to adjust the influence of factors such as age, gender, smoking, lipid level, CHD, and hypertension, the difference becomes statistically insignificant (P* = 0.0212). 82.8% of the participants in the former group suffer from CHD. This is statistically significantly higher than that of the participants in the latter group which is 17.4% (P<0.001; P*<0.001). The average age of the participants with *GALNT2* gene promoter hypermethylation is 62.10±8.21, statistically significantly higher than that of the participants without hypermethylation which is 57.28±9.87 (P = 0.004; P* = 0.008); in the former group, 75.4% of the participants have CHD, significantly higher than 50% in the latter group (P = 0.003; P* = 0.008). As for the *HMGCR* gene, the average age of the participants with promoter hypermethylation is 63.24±8.10, significantly higher than that of the participants without hypermethylation which is 57.79±9.55 (P = 0.003; P* = 0.014); its promoter hypermethylation is likely to be related to smoking. In addition, it seems that the DNA promoter hypermethylation of the three genes is not related to lipid level, hypertension and gender.

**Table 3:** Association of DNA hypermethylationed *ABCG1*, *GALNT2* and *HMGCR* genes with clinic characteristics.

Characteristics	ABCG1				GALNT2				HMGCR			
	U	M	*P*-value	*P*-value^*^	U	M	*P*-value	P-value*	U	M	*P*-value	*P*-value^*^
	(Mean ± s.e.)	(Mean ± s.e.)			(Mean ± s.e.)	(Mean ± s.e.)			(Mean ± s.e.)	(Mean ± s.e.)		
Age(yrs)	57.28±10.01	60.44±9.05	O.O66	0.651	57.28±9.87	62.10±8.21	**0.004**	**0.008**	57.79±9.55	63.24±8.10	**0.003**	**0.014**
Gender (M/F)	25/21	63/30	0.125	0.423	48/30	40/21	0.624	0.971	62/26	36/15	0.987	0.139
Smoking(Y/N)	17/29	47/46	0.132	0.819	34/44	30/31	0.512	0.522	50/48	14/27	0.071	**0.033**
Hypertension(Y/N)	21/25	58/35	0.063	0.413	42/36	37/24	0.422	0.897	55/43	24/17	0.793	0.315
Diabetes(Y/N)	3/43	25/68	**0.010**	0.212	16/62	12/49	0.902	0.144	16/82	12/29	0.087	0.204
CHD(Y/N)	8/38	77/16	**<0.001**	**<0.001**	39/39	46/15	**0.003**	**0.008**	56/42	29/12	0.136	0.459
TC(mmol/l)	4.44±0.99	4.50±1.18	0.735	0.285	4.41±1.20	4.57±0.99	0.401	0.625	4.46±1.16	4.53±1.02	0.762	0.082
TG(mmol/l)	1.60±0.76	1.89±2.73	0.525	0.513	1.86±2.97	1.70±0.78	0.680	0.943	1.82±2.66	1.73±0.84	0.845	0.243
HDL-C(mmol/l)	1.10±0.369	1.10±0.27	0.900	0.252	1.10±0.31	1.10±0.29	0.919	0.861	1.12±0.30	1.05±0.30	0.205	0.071
LDL-C(mmol/l)	2.64±0.77	2.73±1.01	0.615	0.166	2.61±0.97	2.81±0.88	0.198	0.325	2.69±0.94	2.72±0.93	0.863	0.117

M: methylationed; U: unmethylationed; *P*-value*: adjusted for age, gender, smoking (smoker vs never smoker), lipid level, history of hypertension, and history of diabetes by Cox regression. *P*<0.05 is considered statistically significant.

**Figure 2:**
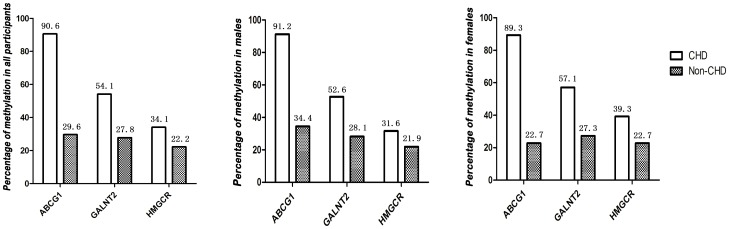
Methylation percent of the *ABCG1*, *GALNT2* and *HMGCR* genes promoter in the CHD cases and Non-CHD controls according to subgroup analysis by total samples and gender.

Furthermore, we then analysed the methylation patterns of *ABCG1*, *GALNT2* and *HMGCR* genes promoter regions in the CHD patients and non-CHD subjects. As shown in Fig. 2, the promoter region of the *ABCG1* gene is hypermethylated in 90.5% of the CHD patients and 29.6% of the non-CHD subjects. Similarly, the promoter regions of the *GALNT2* gene and *HMGCR* gene are hypermethylated in a higher percentage of the CHD patients than the non-CHD subjects (54.1% vs. 27.8% and 34.1% vs. 22.2% respectively). The promoter methylation of the *ABCG1, GALNT2 and HMGCR* genes was detected in 91.2%, 52.6%, and 31.6% of the male CHD patients, while that of the male non-CHD subjects is 34.4%, 28.1%, and 21.9%. In the female study group, the promoter methylation of the three genes was detected in 89.3%, 57.1%, and 39.3% of the CHD patients and 22.7%, 27.2%, and 22.7% of non-CHD subjects respectively.

There was a significant statistical association of the promoter hypermethylation of the *ABCG1* gene with CHD risk (OR = 22.859; 95% CI, 8.989–58.135; p<0.001 and OR = 19.966; 95% CI, 7.319–54.468; *P* ^*^<0.001; *P* ^*^: adjusted for age, gender, smoking, lipid level, hypertension, and diabetes). Hence, *ABCG1* promoter methylation status could be used to predict the risk of CHD in the total study sample. Similar relationship was found between the methylation status of the *GALNT2* gene promoter region and the risk of CHD (OR = 3.067; 95% CI, 1.474–6.380; p = 0.002 and OR = 2.978; 95% CI, 1.335–6.646; *P* ^*^ = 0.008) (Table 4). In contrast, the methylation status of the *HMGCR* gene promoter regions is not shown to be associated with CHD (OR = 1.813; 95% CI, 0.829–3.965; *P*  = 0.134 and OR = 1.388; 95% CI, 0.572–3.371; *P* ^*^ = 0.469). As displayed in Table 4, the *ABCG1* and *GALNT2* gene promoter regions are positively associated with CHD both in the male group (*ABCG1*: OR = 19.855, 95% CI, 6.148–64.119, *P*<0.001; *GALNT2*: OR = 2.840; 95% CI, 1.121–7.194; *P* = 0.025 and *ABCG1*: OR = 16.291, 95% CI, 4.917–53.974, *P* ^*^<0.001; *GALNT2*: OR = 2.717; 95% CI, 1.010–7.309; *P* ^*^ = 0.048) and the female group (*ABCG1*: OR = 28.333, 95% CI, 5.964–134.609, *P*<0.001; *GALNT2*: OR = 3.556; 95% CI, 1.071–11.808; *P* = 0.035 and *ABCG1*: OR = 52.923, 95% CI, 5.329–525.607, *P* ^*^ = 0.001; *GALNT2*: OR = 6.355; 95% CI, 1.248–32.365; *P* ^*^ = 0.026). The analysis in the two gender groups demonstrates both male- and female-dependent effects of the *ABCG1* and *GALNT2* gene promoter methylation status in prediction of CHD. However, the statistical evidence is not high enough to support an association between the *HMGCR* gene promoter methylation and the CHD in either of the gender group (Table 4).

**Table 4:** Methylation status of the *ABCG1*, *GALNT2* and *HMGCR* genes promoter in the CHD cases and Non-CHD controls according to subgroup analysis by total samples and gender.

Gene	Methylation status	CHD	Non-CHD	*P*-value	OR(95%CI)	*P*-value^*^	OR(95%CI)^*^
Total samples							
ABCG1	Methylated	77	16	**<0.001**	22.859(8.989–58.135)	**<0.001**	19.966(7.319–54.468)
	Unmethylated	8	38				
GALNT2	Methylated	46	15	**0.002**	3.067(1.474–6.380)	**0.008**	2.978(1.335–6.646)
	Unmethylated	39	39				
HMGCR	Methylated	29	12	0.134	1.813(0.829–3.965)	0.469	1.388(0.572–3.371)
	Unmethylated	56	42				
Sex(Male)							
ABCG1	Methylated	52	11	**<0.001**	19.855(6.148–64.119)	**<0.001**	16.291(4.917–53.974)
	Unmethylated	5	21				
GALNT2	Methylated	30	9	**0.025**	2.840(1.121–7.194)	**0.048**	2.717(1.010–7.309)
	Unmethylated	27	23				
HMGCR	Methylated	18	7	0.328	1.648(0.602–4.513)	**0.841**	0.896(0.307–2.613)
	Unmethylated	39	25				
Sex(Female)							
ABCG1	Methylated	25	5	**<0.001**	28.333(5.964–134.609)	**0.001**	52.923(5.329–525.607)
	Unmethylated	3	17				
GALNT2	Methylated	16	6	**0.035**	3.556(1.071–11.808)	**0.026**	6.355(1.248–32.365)
	Unmethylated	12	16				
HMGCR	Methylated	11	5	0.213	2.200(0.629–7.700)	0.307	2.483(0.433–14.237)
	Unmethylated	17	17				

OR: odds ratio; CI: confidence interval; *P*-value: probability from the Pearson Chi-Square exact test comparing the methylation status for CHD.

Cases and Non-CHD controls; *P*-value*: adjusted for age, gender, smoking (smoker vs never smoker), lipid level, history of hypertension, and history of diabetes by Cox regression. *P*<0.05 is considered statistically significant.

## Discussion

Coronary heart disease is one of the most prevalent and preventable health problems that cause high morbidity and mortality in both the developed and developing countries worldwide [24]. High level of the circulating LDL-c and low level of the HDL-c are strong risk factors for CHD [25].

*ABCG1* belongs to the *ABCG* family of reverse half transporters. Similar to *ABCA1*, *ABCG1* exports excess cellular cholesterol into the HDL pathway and reduces cholesterol accumulation in the macrophages [16]. Multiple potential transcripts of human *ABCG1* that use alternate exons or promoters have been identified and found to play an important role in the transportation of dietary lipid components [26].

Another strong candidate for the HDL-c regulation in humans is *GALNT2*, which regulates O-linked oligosaccharide biosynthesis [27]. *GALNT2* mutation has been reported to underlie non-sialyation of *APOC3*, which in turn leads to the increased LPL activity in humans [28]. LDL-c levels are significantly associated with GWAS SNPs near HMG Co-A reductase (*HMGCR*), the rate-limiting enzyme for cholesterol biosynthesis [29]. Typical of GWAS-identified variants, an LDL-associated genetic variant near *HMGCR* has an allele frequency of 39% and influences LDL cholesterol levels by a modest 2.5 mg/dL. However, use of statins, which inhibit the function of the rate-limiting enzyme, encoded by *HMGCR*, in the cholesterol synthesis, typically decreases LDL cholesterol levels by 20–40%, or ∼14–70 mg/dL [30].

Sharma P et al has provided evidence that there is a potential relationship between the global DNA hypomethylation and locus-specific hypermethylation in the process of atherosclerosis, which has yet to be explored [31]. Promoter CpG island hypermethylation is closely related to gene inactivation and silencing, resulting in loss of expression of tumour suppressor genes and X-chromosome inactivation [32]–[33]. Aberrant promoter region methylation of tumour-suppressor genes is associated with the mechanism for carcinogenesis. Altered gene expression and cell proliferation in atherosclerotic lesions have some similar characteristics of certain solid tumours and thus might share similar mechanisms that lead to CHD [34]. Indeed, it is conceivable that epigenetic modifications, especially alteration in DNA methylation status, are increasingly being recognized as a key factor in the pathogenesis of complex disorders, including atherosclerosis [9].

Several studies have separately shown that methylation of CpG islands of the CHD risk genes has a significant role in the development of CHD. Simon et al found that epigenetic changes within the *ABCA1* gene promoter contribute to the inter-individual variability in plasma HDL-C concentrations and are associated with CHD expression [12]. For example, the modulation of methylation-induced FVIIa concentrations was observed only in A1A1, where the higher methylation status resulting in lower FVIIa being more prevalent within the CHD-free group compared to the CHD group (p = 0.011) [14]. *PLA2G7* methylation might exert its effects on the risk of CHD by regulating the levels of TC, TG, and ApoB in females. The gender disparities in the *PLA2G7* methylation may have an effect in the molecular mechanisms underlying the pathophysiology of CHD [11]. Methylation associated inactivation of the *ER*, a gene in vascular tissue, may influence atherogenesis and aging of the vascular system [9]. These examples demonstrate that DNA aberrant methylation potentially play a predominant role in the pathogenesis of cardiovascular disease.

In the present study, methylation status of the promoter region of the CHD risk genes *ABCG1*, *GALNT2* and *HMGCR* were firstly investigated. Our study found a significantly higher promoter methylation of *ABCG1* and *GALNT2* in the CHD group than in the non-CHD group. The promoter region of the *ABCG1*, *GALNT2* and *HMGCR* genes was hypermethylated in 90.5%, 54.1% and 34.1% of CHD patients and 29.6%, 27.8% and 22.2% of non-CHD subjects. There is statistically significant evidence to show that the *ABCG1* gene promoter hypermethylation increases the risk of CHD in the total samples (OR = 22.859; 95% CI, 8.989–58.135; *P*<0.001 and OR = 19.966; 95% CI, 7.319–54.468; *P* ^*^<0.001). Similar results were obtained for the *GALNT2* gene (OR = 3.067; 95% CI, 1.474–6.380; *P* = 0.002 and OR = 2.978; 95% CI, 1.335–6.646; *P* ^*^ = 0.008). However, we found no convincing association between the DNA methylation of *HMGCR* gene promoter and CHD risk. The promoter methylation of *ABCG1* and *GALNT2* genes are significantly positively associated with CHD risk both in the male and the female groups. In addition, we found a significant association between the promoter methylation status of the three genes and several clinical characteristics. This study found that 82.8% of the participants with *ABCG1* gene promoter hypermethylation have CHD, while only 17.4% of the participants without hypermethylation have it; the percentage of the participants have diabetes is 26.9% in the former group and 6.5% in the latter group. The average age of the participants with *GALNT2* gene promoter hypermethylation is 62.10±8.21, while that of the participants without hypermethylation is 57.28±9.87; in the former group, 75.4% of the participants have CHD, compared to only 50% in the latter group. As for the *HMGCR* gene, the average age of the participants with promoter hypermethylation is 63.24±8.10 and that of the participants without hypermethylation is 57.79±9.55; its promoter hypermethylation is likely to be related to smoking. In the past decade, several clinical research studies have been focused on the molecular mechanisms of aberrant DNA methylation in the development of CHD, but our current understanding into these processes is limit.

There are some limitations in our study. Firstly, to the best of our knowledge, this is the first study to analyse the involvement of the *ABCG1*, *GALNT2* and *HMGCR* genes promoter methylation in CHD. The sample size in our study is comparatively small, as only 85 CHD patients and 54 participants without CHD were recruited. Hence, further replication studies with larger sample size are required to confirm our findings. Secondly, as our study only recruited Han Chinese people, further replication studies of the relationship between the promoter methylation of the three genes and the risk of CHD in other different ethnic populations are needed. In addition, our study was only designed to determine whether the DNA methylation status of the three genes' promoter regions has a predominant role in the development of CHD. The underlying mechanisms have not been scrutinised. Aberrant methylation status of these genes could contribute to CHD risk via manipulating gene expression, affecting protein levels, influencing blood lipid levels, or even participating in the pathogenesis of atherosclerosis. Therefore, the relationship between DNA methylation status and gene expression regulation will have to be tested in further studies.

In conclusion, the present work has provided supportive evidence to the link between DNA methylation status and the cardiovascular risk profile. Our data indicated that the *ABCG1* and *GALNT2* gene promoter hypermethylation increases the risk of CHD. However, no convincing association between that of the *HMGCR* gene and CHD risk was found. CHD, smoking and aging are likely to be the important factors influencing DNA hypermethylation. The aforementioned results can potentially help improve the current clinical diagnosis and treatment of CHD. For a better understanding of the pathophysiological processes of CHD, future studies are required to investigate the relationship between DNA methylation and gene expression regulation.
